# Supplementary material for: Empirical methods for controlling false positives and estimating confidence in ChIP-Seq peaks
Source: BMC Bioinformatics. 2008 Dec 5;9:523. doi: 10.1186/1471-2105-9-523 (PMC2628906; doi:10.1186/1471-2105-9-523)
Supplement: Additional File 1 — A variety of html documents from the USeq web site detailing the available applications, their best usage, output file type descriptions, command line menus, etc. [file 1471-2105-9-523-S1.zip › USeqUserGuides/JavaDoc.zip!/JavaDoc/util/bio/annotation/GenericFeature.html]

GenericFeature


|  |  |  |
| --- | --- | --- |
| **Overview** \| **Package** \| **Class** \| **Use** \| **Tree** \| **Deprecated** \| **Index** \| **Help** | |  |
| **PREV CLASS**   **NEXT CLASS** | **FRAMES**    **NO FRAMES**     **All Classes** |  |
| SUMMARY: NESTED \| FIELD \| CONSTR \| METHOD | DETAIL: FIELD \| CONSTR \| METHOD |  |


---


## util.bio.annotation Class GenericFeature

```
java.lang.Object
  util.bio.annotation.GenericFeature
```

**All Implemented Interfaces:**: java.lang.Comparable

---

``` public class GenericFeature extends java.lang.Object implements java.lang.Comparable ```

Class to hold user defined gff items like CRMs, enhancers, unpublished single track notation.

---

**Constructor Summary**

`GenericFeature(Gff3Feature f)`

`GenericFeature(GffFeature gffFeature)`


| **Method Summary** | |
| --- | --- |
| `int` | `compareTo(java.lang.Object other)` |
| `java.lang.String` | `getAttributes()` |
| `int` | `getEnd()` |
| `java.lang.String` | `getFeatureType()` |
| `java.lang.String` | `getName()` |
| `int` | `getOrientation()` |
| `double` | `getScore()` |
| `int` | `getStart()` |
| `java.lang.String` | `getStrand()` |
| `int` | `getTrackNumber()` |
| `void` | `setTrackNumber(int i)` |

**Methods inherited from class java.lang.Object**

`equals, getClass, hashCode, notify, notifyAll, toString, wait, wait, wait`

**Constructor Detail**

### GenericFeature

```
public GenericFeature(GffFeature gffFeature)
```

---


### GenericFeature

```
public GenericFeature(Gff3Feature f)
```


**Method Detail**

### compareTo

```
public int compareTo(java.lang.Object other)
```

:   **Specified by:**: `compareTo` in interface `java.lang.Comparable`

---


### getFeatureType

```
public java.lang.String getFeatureType()
```

---


### getOrientation

```
public int getOrientation()
```

---


### getTrackNumber

```
public int getTrackNumber()
```

---


### setTrackNumber

```
public void setTrackNumber(int i)
```

---


### getName

```
public java.lang.String getName()
```

---


### getScore

```
public double getScore()
```

---


### getAttributes

```
public java.lang.String getAttributes()
```

---


### getStrand

```
public java.lang.String getStrand()
```

---


### getEnd

```
public int getEnd()
```

---


### getStart

```
public int getStart()
```


---


|  |  |  |
| --- | --- | --- |
| **Overview** \| **Package** \| **Class** \| **Use** \| **Tree** \| **Deprecated** \| **Index** \| **Help** | |  |
| **PREV CLASS**   **NEXT CLASS** | **FRAMES**    **NO FRAMES**     **All Classes** |  |
| SUMMARY: NESTED \| FIELD \| CONSTR \| METHOD | DETAIL: FIELD \| CONSTR \| METHOD |  |


---
